# An Efficient Chaos-Based Image Encryption Technique Using Bitplane Decay and Genetic Operators

**DOI:** 10.3390/s22208044

**Published:** 2022-10-21

**Authors:** Ramesh Premkumar, Miroslav Mahdal, Muniyandy Elangovan

**Affiliations:** 1 Department of Electronics and Communication Engineering, Mount Zion College of Engineering and Technology, Pudukottai 622 507, India; 2 Department of Control Systems and Instrumentation, Faculty of Mechanical Engineering, VSB-Technical University of Ostrava, 17. Listopadu 2172/15, 708 00 Ostrava, Czech Republic; 3 Department of R&D, Bond Marine Consultancy, London EC1V 2NX, UK

**Keywords:** bitplane slicing, block swapping, mutation, scrambling, logistic map, CCI map

## Abstract

Social networks have greatly expanded in the last ten years the need for sharing multimedia data. However, on open networks such as the Internet, where security is frequently compromised, it is simple for eavesdroppers to approach the actual contents without much difficulty. Researchers have created a variety of encryption methods to strengthen the security of this transmission and make it difficult for eavesdroppers to get genuine data. However, these conventional approaches increase computing costs and communication overhead and do not offer protection against fresh threats. The problems with current algorithms encourage academics to further investigate the subject and suggest new algorithms that are more effective than current methods, that reduce overhead, and which are equipped with features needed by next-generation multimedia networks. In this paper, a genetic operator-based encryption method for multimedia security is proposed. It has been noted that the proposed algorithm produces improved key strength results. The investigations using attacks on data loss, differential assaults, statistical attacks, and brute force attacks show that the encryption technique suggested has improved security performance. It focuses on two techniques, bitplane slicing and followed by block segmentation and scrambling. The suggested method first divides the plaintext picture into several blocks, which is then followed by block swapping done by the genetic operator used to combine the genetic information of two different images to generate new offspring. The key stream is produced from an iterative chaotic map with infinite collapse (ICMIC). Based on a close-loop modulation coupling (CMC) approach, a three-dimensional hyperchaotic ICMIC modulation map is proposed. By using a hybrid model of multidirectional circular permutation with this map, a brand-new colour image encryption algorithm is created. In this approach, a multidirectional circular permutation is used to disrupt the image’s pixel placements, and genetic operations are used to replace the pixel values. According to simulation findings and security research, the technique can fend off brute-force, statistical, differential, known-plaintext, and chosen-plaintext assaults, and has a strong key sensitivity.

## 1. Introduction

The rapid development of systems and information-sharing technology has enabled the transmission and storage of an ever-increasing amount of multimedia content, such as images and movies. Innovative image encryption techniques have been suggested to increase the security of these photos, as there has been a greater emphasis on the security of cutting-edge information, including in pictures.

Both substitution and permutation are crucial systems with high levels of redundancy and close connections. Substitution makes it impossible to check the content of the figure for repetitions and quantitative examples, since it makes the relationship between the key and the content of the figure more complex. However, permutation reduces repetition by increasing the quantity of plaintext above the overall content of the image. Even though each of these strategies stands alone and is highly vulnerable to attack, when combined, they frequently offer extraordinary levels of security. Standard encryption techniques such as the DES (Data Encryption Standard), IDEA (International Data Encryption Algorithm), and RSA (Rivest–Shamir–Adleman) cannot be used on images due to their particular properties, such as a high pixel density and a big information limit [1]. Row-, column-, and pixel-shuffled permutations in three stages are supported on RGB (red, green, and blue) in [2]. Different techniques, such as XOR (exclusively-OR) and S boxes, are used for permutation and diffusion [3]. The information thrashing in the frequency domain technique employed by the genetic algorithm is described in [4]. Ciphered information was inserted into the occurrence coefficients that define the spatial domain picture limitations. As a result, the envelop image changed the least and had the most compatibility with each visual system. According to [5], one of the main advantages of using the GA (genetic algorithm) for segmentation is its capacity to select a few parameters, such as the size of the inspection window or some heuristic edges, or the ideal number of regions of a division result. In the paper by [6], an algorithm was presented that integrates adjacent sequence and borderline alteration for figure division applications, as well as the bitplane possibility representation based on agglomerative fuzzy computation. Moreover, other encryption schemes, such as four-picture encryption, encryption employing gesture broadcast, and encryption using original image uniqueness, are provided in [7]. Coupled map lattices are recommended in the genetic algorithm [8] to speed up convergence. Kalita et al. [9,10,11] explain several crossover and mutation strategies as well as genetic algorithms. In the work of [12], bitplanes are used to swap and permute the binary components of the image. In [13], which has three models and is a hybrid, they used several diffusion models. In their work, [14] offered a multilevel picture technique based on a q-chaos map and light sampling, replacing images randomly using discrete wave transmission technology. Fractional Fourier transform was used in [15] to increase security.

The Baker map and CLM (compound logistic map) are used to achieve permutation and substitution in this case. The hybrid GA is available in [16], and it includes one-point cross and mutation phases. They represent the key stages of a genetic algorithm. In [17], the authors offered a permutation strategy based on grey code. In [18], the authors putrefied the first image into eight biplanes and offered one of the practical justifications for low control, high rate, and genuine time secure multimedia transmission. With the chaos-based pseudo-arbitrary twofold number generator’s keystream evolution, only the necessary piece planes are scrambled [18]. Blocks are switched over in the block permutation image in [19] using a separate block image, whose locality is determined using the Logistic–Sine system. Block diffusion is the method of removing bordering pixels and the secret keys with the least amount of variance possible, resulting in a significant variation in the encrypted images. To achieve accurate segmentation, bitplanes are based on the segmentation method in [20]. The binary bitplane approach combines three bitplane methods. Here, calculations encrypt images using the bitplane of an image as the defence key bitplane [21]. This arrangement can be applied to genuine communication and offers scrambling and diffusion with environment alternation [22]. The authors of [23] offer circular shifts by rows, columns, and logical operators, whereby PCA, LBP, GWO, and KELM are combined to classify hyperspectral images in this research. PLG-KELM enhances small-sample classification and generalisation [24]. In DenseNet, there are problems of insufficient feature utilisation, a single type of convolution kernel, and incomplete network optimisation. Hybrid dilated convolution (HDC) is a new dilated convolution method that improves on previous methods [25]. Yao et al. [26] developed scale-adaptive mathematical morphology spectrum entropy to improve scale selection. Deng et al. [27] showed that the vibration signal can be decomposed into a set of intrinsic mode functions (IMFs) by using the empirical mode decomposition method. The fuzzy information entropy values of the IMFs are calculated to reveal the intrinsic characteristics of the vibration signal. The improved PSO algorithm can effectively improve the classification accuracy of the LS-SVM. Wu and Wu [28] argued that the TDGVRPSTW approach has a faster convergence speed and better optimisation ability than the comparison method. The geographical distribution of customers will affect the proportion of each part in the total cost. Enterprises should have a reasonable estimate of the time sensitivity of fresh agricultural products to be distributed. Wu and Wu [29] presented a study on the split delivery vehicle routing problem that arises in the distribution of fresh agricultural products. A variable neighbourhood search combined with the nondominated sorting genetic algorithm II (VNS-NSGA-II) and techniques for order preference by similarity to an ideal solution (TOPSIS) were applied. Arthi et al. [30] developed a cryptosystem based on the 4D Lorenz type hyperchaos and deoxyribonucleic acid (DNA) encoding mechanism. Various performance metrics were analysed for several images and the experimental results showed that the proposed scheme was effective against brute force attacks.

Kapinesh et al. [31] developed an efficient algorithm for image encryption. The technique consists of many levels of encryption for securely transmitting data that is vulnerable to cyberattacks. Images are encrypted by applying the generated sequence key to the original image, which is further encrypted using various mathematical computations. De Dieu et al. [32] developed a new image encryption scheme based jointly on DNA coding and the chaotic sequence generated by a new 3D chaotic system without linear terms. Security tests, such as statistical and differential analysis, occlusion, and data loss attacks, as well as brute force attacks, proved the efficiency and security of the proposed cryptosystem. Ramadoss et al. [33] designed a novel chaotic circuit with a symmetrical nonlinear component by replacing the single semiconductor diode in the original circuit. The novel oscillator exhibited more interesting dynamical properties, including, for instance, symmetry-breaking bifurcation, merging crisis, and coexisting multiple attractors. Nestor et al. [34] developed an image cryptosystem using the randomness of a hyperchaotic system with hyperbolic nonlinearity and permutation and substitution operations. The SHA-256 hash value of the original image was used to generate the secret key, which renders chosen/known-plaintext attacks impossible.

In 1971, Prof. L. Chua introduced a new circuit element which exhibited a different behaviour from that displayed by any of the three known passive elements: the resistor, the capacitor, or the inductor. Since then, the nonlinear and nonvolatile properties of memcapacitors and meminductors have attracted the interest of many researchers [35]. Laser-lithographed graphene oxide memristors are surface-fabricated through a graphene oxide coating on a polyethene terephthalate substrate. When the laser fluence is appropriately tuned during the fabrication process, the devices present a characteristic pinched closed-loop in the current–voltage relation, revealing the unique fingerprint of the memristive hysteresis [36]. Hao et al. [37] proposed a novel colour image encryption algorithm based on the fractional order laser chaotic system and DNA mutation. The algorithm showed strong encryption capabilities that could withstand multiple decryption methods. Ramakrishnan et al. [38] stated that the AJJJOCIT derived from a resistive capacitive-shunted JJ model with a cosine interference term has two or no equilibrium points as a function of the direct current (DC). One of the equilibrium points is unconditionally unstable and the other equilibrium point has a Hopf bifurcation, where its expression depends on the DC and coherence parameters. Qiu et al. [39] proposed a Rubik’s Cube scrambling method to scramble a three-dimensional bit-level matrix of the colour image directly. Shang et al. [40] presented an efficient video encryption scheme by employing a chaotic cypher. First, selective encryption was completed by encrypting the fixed-length codeword. Then, for each frame, macroblock shuffling was executed in the video bit stream using a chaos-based permutation. The proposed chaotic system employed two of the simplest chaotic maps as well as avoided the floating-point arithmetic.

For both text and image encryption, Signing et al. [41] suggested a cryptosystem based on the unique randomness created by a chaotic Jerk system with DNA coding and a hump structure. Before diving deeper into the phenomenon and putting the encryption method into practice, it is helpful to do some preliminary research on the dynamic features of a Jerk system and DNA coding. Liu et al. [42] proposed a DNA-encoded logistic map and spatial map-based colour image encryption technique. The algorithm begins with the R, G, and B channels being scrambled using a logistic map. The spatial map is then used to control an XOR operation between the pixel channels and a sequence matrix. Ravichandran et al. [43] proposed two stages of shuffling and one level of dissemination. Pixels are initially shuffled in rows and columns, and then in blocks, constituting the first stage of the shuffling process. Yaghouti and Moattar [44] applied the Chen chaotic system to generate random sequences, from which various arrays are constructed for use in image permutation and key stream generation. Using a 3D logistic map, DNA encoding rules were randomly selected to encode plain picture blocks. Lone et al. [45] presented a novel algorithm for image encryption by invoking the well-known deoxyribonucleic acid (DNA) method and 3D chaos maps. The efficiency of the proposed algorithm was verified via a series of experiments carried out on some test images.

The main issue is that the existing system’s computation time is high due to a large number of crucial factors. The new image cipher technique for image data conducts both encryption and decryption for better security in response to this vulnerability. The following contributions are made by this work:

- The proposed novel encryption scheme is based on the development of confusion–diffusion methods.
- It introduces a revolutionary compound crossover technique.
- A unique compound mutation technique is being developed.
- Incorporating a new 3D CCI (Compound Cubic, Circle, and ICMIC) map provides for key stream creation.
- Strong security and a sizable speed improvement are provided by the proposed encryption system.
- Image encryption has a wide range of applications and greatly aids in data encryption. A strong image encryption approach is employed to achieve decent performance. Two different kinds of image data sets are employed to evaluate this method. Two combined techniques are employed in this strategy to produce strong encryption performance.

This paper presents a genetic operator-based image encryption technique. In the initial stage, original colour photographs are broken up into blocks, continually randomly mixed, and then dispersed across eight planes. During the crossover stage, eight planes from two picture blocks are randomly exchanged and mixed. In the final stage, a 3D compound cubic, circle, and ICMIC (CCI) map are used to construct the key stream. For the encryption, hybrid operators and a secret key are used.

## 2. Proposed System Design

**Figure 1:**
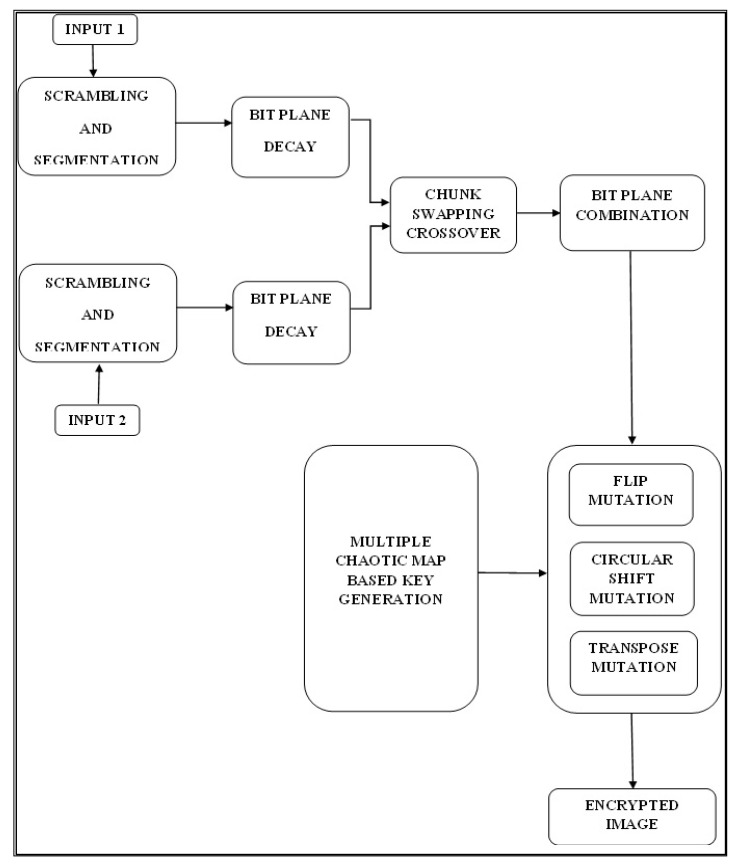
Proposed system design for encryption.

This section demonstrates the efficacy of a genetic operator-based image encryption technique for multimedia security (Figure 1). The key stages of this strategy are chunk swapping crossover and hybrid mutation. The proposed design input contains two images. The initial stage involves segmenting and individually scrambling each image. These chaotic images are then again randomly split into eight-bit planes using the bitplane slicing algorithm. The deconstructed bitplanes are then randomly switched using the genetic crossover operator. This crossing is referred to as block switching, and the bitplanes are then combined afterwards. Block switching raises the entropy of the system, making it more secure. The combined bitplane values in pixels are altered by the mutation operator. In this instance, three mutation operations are merged: transpose mutation, flip mutation, and circular shift mutation.

### 2.1. Step 1 Scrambling and Segmentation

**Figure 2:**
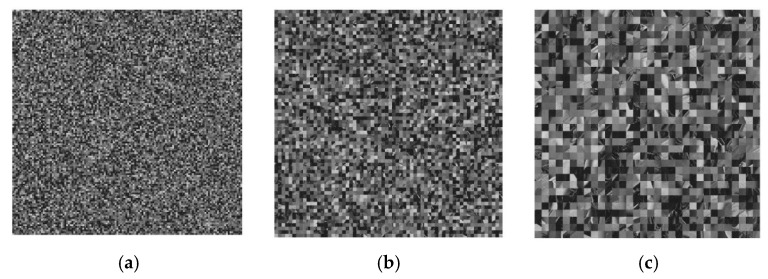
Scrambling and segmentation. Scrambling with a size of (**a**) 8 × 8; (**b**) 16 × 16; (**c**) 32 × 32.

Image division, an important step in advanced image management, is crucial for further image recognition, analysis, and comprehension of the key edges, areas, surfaces, and other picture features [21]. Utilising a single block size is the simplest kind of block segmentation. First, the entire figure is divided into a lattice of blocks, and then each block is classified using a certain attribute. In this case, several kinds of matrix blocks to complete the scrambling are used, which is the random change of the pixel position. Figure 2 depicts them.

### 2.2. Step 2 Bitplane Slicing

**Figure 3:**
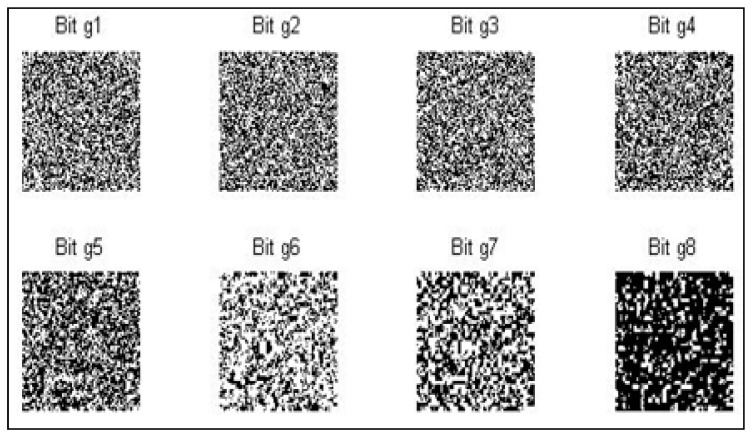
The output of a scrambled image.

The initial image degrades into several binary images. Consider how the bitplanes in the Figure 3 are arranged in an 8 to 1 array from bitplane 1 to bitplane 0 (LSB) to bitplane 7. (MSB). In the case of 8-bit bytes, plane 0 encloses all low-order bits, including the pixels in the image, and plane 7 encloses all high-order bits.

(1) $$N=\overset{7}{\underset{i=0}{\sum }}B_{i}2^{i-1}=B_{0}2^{-1}+B_{1}2^{0}\ldots \ldots .+B_{7}2^{6}$$

### 2.3. Step 3 Chunk Swapping Crossover Operation

**Figure 4:**
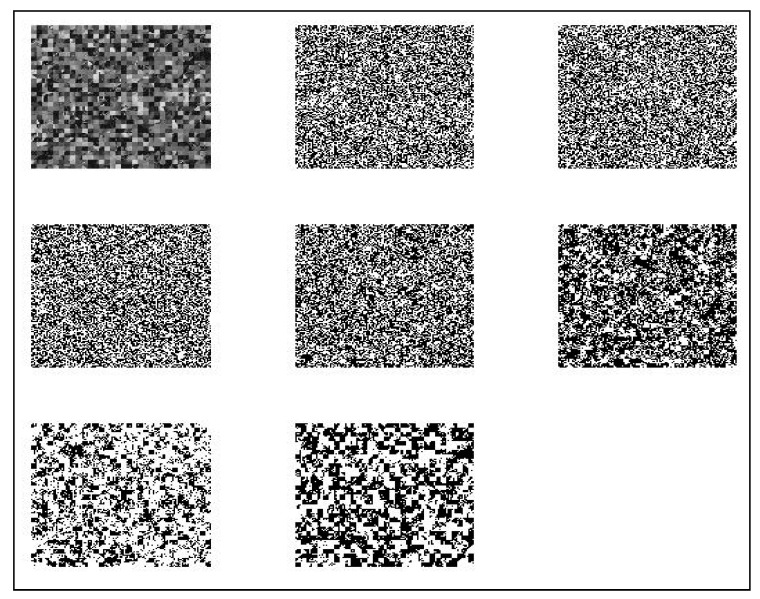
The output of a grey code-based bitplane slicing.

Using two chromosomes, GA is used in this instance for crossover and mutation. To create the encrypted image, mutations involving block swapping, crossover and flip, transposition, and circular shift are used. To check the encryption process for deviation, mutation speed is frequently difficult. Every other bit in the thread is flipped and the mutation time is set to a charge of greater than 0.5. Intentional jobs for crossover and mutation are used in histogram uniformity. First, block swapping at the crossover stage is performed, which involves switching the blocks of the bitplanes (Figure 4).

### 2.4. Step 4 Hybrid Mutation Operation

**Figure 5:**
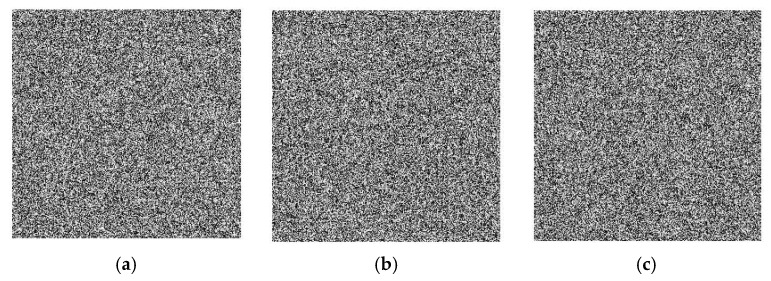
The output of mutation: (**a**) circular shift; (**b**) transpose; (**c**) flip mutation.

The hybrid mutation is carried out at the mutation stage. They are the circular shift, flip, and transpose (Figure 5).

### 2.5. Step 5 Combined Keystream

The 3D combined cubic, circle, and ICMIC (CCI) maps show the chaotic utilisation when the control parameters A is in the interval [0, 45] and the value of b is set between (0, ∞). The CCI map is given by

x(n + 1) = mod((m * x(n) * (1 − (x(n))^2)) + (mod(x(n) + b − (a/(2 * pi)) * sin(2 * pi * x(n)),1)) + (sin(r/x(n))),1);
y(n + 1) = mod((m * y(n) * (1 − (y(n))^2)) + (mod(y(n) + b − (a/(2 * pi)) * sin(2 * pi * y(n)),1)) + (sin(r/y(n))),1);
z(n + 1) = mod((m * z(n) * (1 − (z(n))^2)) + (mod(z(n) + b − a/(2 * pi)) * sin(2 * pi * z(n)),1)) + (sin(r/z(n))),1);(2)

**Figure 6:**
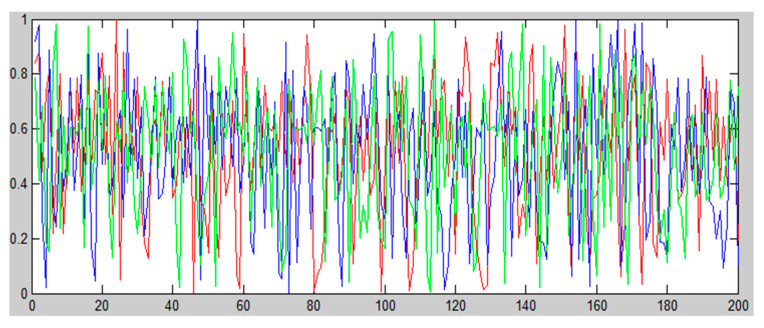
3D CCI map with 200 iterations.

The key matrix was generated by cubic, circle, and ICMIC. The produced new key stream was mixed with the mutation process. The key x combined with boundary mutation with the logical operation. Then, key y was combined with nonuniform mutation and key z was combined with uniform mutation by logical operation. After that, three new encrypted images were produced. Compound the three encrypted images to produce a single encrypted image to increase the complexity of the algorithm. Figure 6 shows the chaotic utilisation of the 3D CCI map. It seems to produce a more complex set of random keys, which will work to confuse and diffuse the better encryption system. After combining the key with the hybrid mutation process, the result is an encrypted image.

### 2.6. Step 6 Decryption Process

The decryption operation performed the inversion of the encryption process. Initially, execute an inverse hybrid mutation operation using the generated key on the encrypted image. Use the bitplane decomposition method to break the encrypted image down into 8-bit planes. Reverse the bitplanes’ initial order. Reverse scramble the data, then combine all the split pieces. Finally, the decrypted image is the output’s final result.

## 3. Results and Discussion

In this section, the qualitative and quantitative analysis of the performance of the proposed technique is presented and compared with that of the existing methods. The quantitative performance of the proposed technique is demonstrated using several types of analysis, such as statistical analysis, differential analysis, speed analysis, and information entropy.

In this research, experiments on colour images that are commonly used by the research community for comparing the performance analysis of various image processing algorithms are conducted. The test images used in this experiment include Lena, Baboon, Barbara, Monalisa, Peppers, Gold Hills, Fruits, Art, Beetroot, Soccer, Tulips, Flowers, Monarch, and Boat. All 14 of these images are RGB colour images and have different sizes. The sizes of these images vary from 256 × 256 × 3 to 512 × 512 × 3 pixels.

The experiment is carried out on a computing system consisting of one GPU and two CPUs. The mainframe is equipped with an Intel Pentium I CPU running at 2.10 GHz, having 2 GB RAM, and running on the Microsoft Windows 7 operating system. MATLAB 2010a software is used for the simulation.

**Figure 7:**
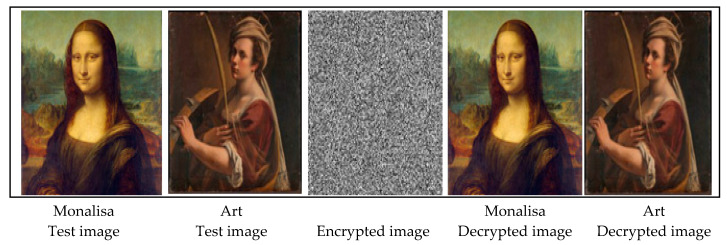

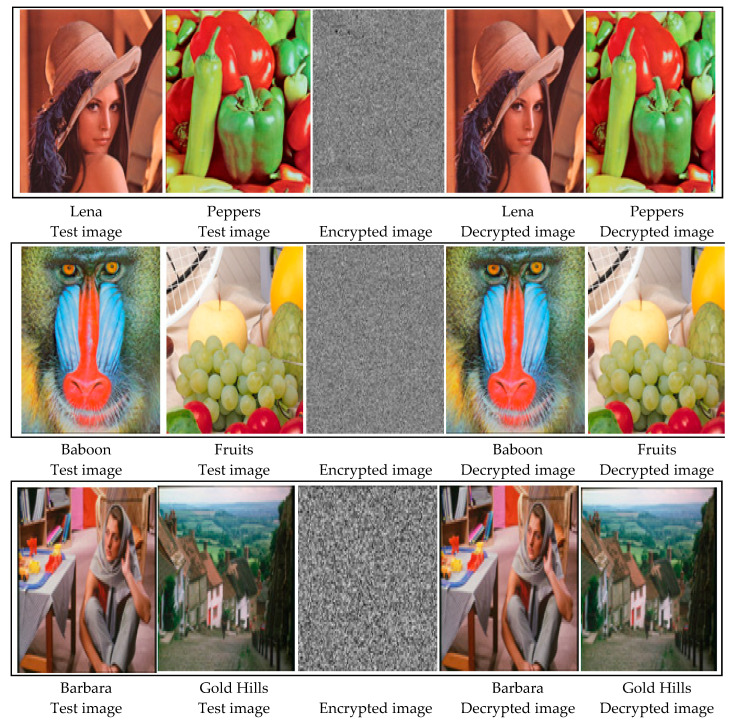
Qualitative analysis of the proposed approach using eight test images. Every row consists of five images. The first two images in each row are test images, the third image represents an encrypted image, and the last two images are the decrypted images corresponding to the test images.

The efficacy of the proposed approach using qualitative analysis is presented in Figure 7. For brevity, only 8 of the total 14 test images are included. The first row of Figure 7 consists of images of paintings created by artists. Images of paintings are characterised by smooth texture regions drawn using different shades of a few colours. It can be seen that the encrypted image (displayed in the middle column) does not reveal any information present in the test image. The smoothness of the texture regions is not affected by the forward and reverse processes of the algorithm. In addition, the algorithm has not disturbed the shades of the colours in the images. Thus, the quality of the decrypted image is not degraded.

The ‘Lena’ and ‘Peppers’ are the most widely used test images in image processing experiments. The reason behind the popularity of these images is that these images contain a nice mixture of edges, smooth regions, shading, texture, and natural objects. Such images help a researcher test and analyse various image processing algorithms. The results of the current approach to these images are presented in the second row of Figure 7. Looking at the encrypted image, it is impossible to get any idea about the variety of content such as edges, smooth regions, shading, and texture present in the test image. However, in the decrypted image, all types of content are reconstructed without any distortion.

Often, applying algorithms to colour images produce output images with unnatural appearances and visually disturbing colour artefacts. In colour images, the relationships among the different colours are not linear. For image reconstruction with original colours, it is necessary to ensure that the nonlinear relationships among the colours are preserved. To assess the performance of the proposed algorithm on a wide spectrum of visible colours, experiments on colour images are carried out. Both the ‘Baboon’ and ‘Fruit’ images displayed in the third row of Figure 7 contain regions of wide colour ranges. It can be seen that the encrypted image does not disclose any information about the colours present in the test image. In the decrypted image, it is seen that all colour details are well preserved and the images have the same natural appearances as that of the test image. Further, it can be noticed that no colour artefacts are introduced in the decrypted image.

The performance of the approach on the images containing man-made objects as well as natural objects is illustrated in the last row of Figure 7. The man-made objects such as the table, rack, and chair are present around the lady in the ‘Barbara’ image. Similarly, in the ‘Gold Hill’ image, a series of houses below natural green fields in the top region can be seen. Man-made objects have shaped edges in the form of straight lines as opposed to the arbitrarily curved smooth and diffusing edges in natural objects. The encrypted image consists of a randomised region in which there is no visible clue about the edges in man-made objects and the smooth regions in natural objects. On the other hand, the details of smooth regions, and all types of edges, are reconstructed without any distortion in the decrypted images. All minute details are visible in the decrypted images.

The qualitative analysis presented in Figure 7 reveals that the proposed algorithm produces encrypted images that possess noise-like characteristics and appear random. The test images and encrypted images are radically different, with no visual clue about the connection between them. This indicates that the algorithm offers good confidentiality. The proposed approach exhibits the ability to reconstruct the decrypted images without introducing any noticeable distortion edges, smooth regions, colours, and textures in the images containing natural objects as well as man-made objects.

### 3.1. Statistical Analysis

The ability of an encryption algorithm to resist potential attacks on encrypted signals is the fundamental requirement of the algorithm. Among the several types of attacks, the statistical attack refers to attacks that exploit the statistical weaknesses of the algorithm. The algorithms that cannot produce truly random numbers are vulnerable to potential statistical attacks. The encryption algorithm should be strong enough to mitigate statistical attacks. The encryption strength of the proposed algorithms is validated toward statistical attacks using histogram analysis (HA) and correlation analysis (CA).

#### 3.1.1. Histogram Analysis

The histogram of an image represents the distribution of pixel values in an image. Common shapes of histograms include Gaussian, bimodal, skewed right, skewed left, uniform, and random distribution. Since the shape of the histogram distribution contains statistical information, statistical attacks can be used to extract the information about the image under attack. A small amount of the sequence may be leaked by the numerical assault if it is not sufficiently smooth. The uniform distribution provides little information about the image. A high-quality encryption algorithm produces images with uniform histogram distribution.

**Figure 8:**
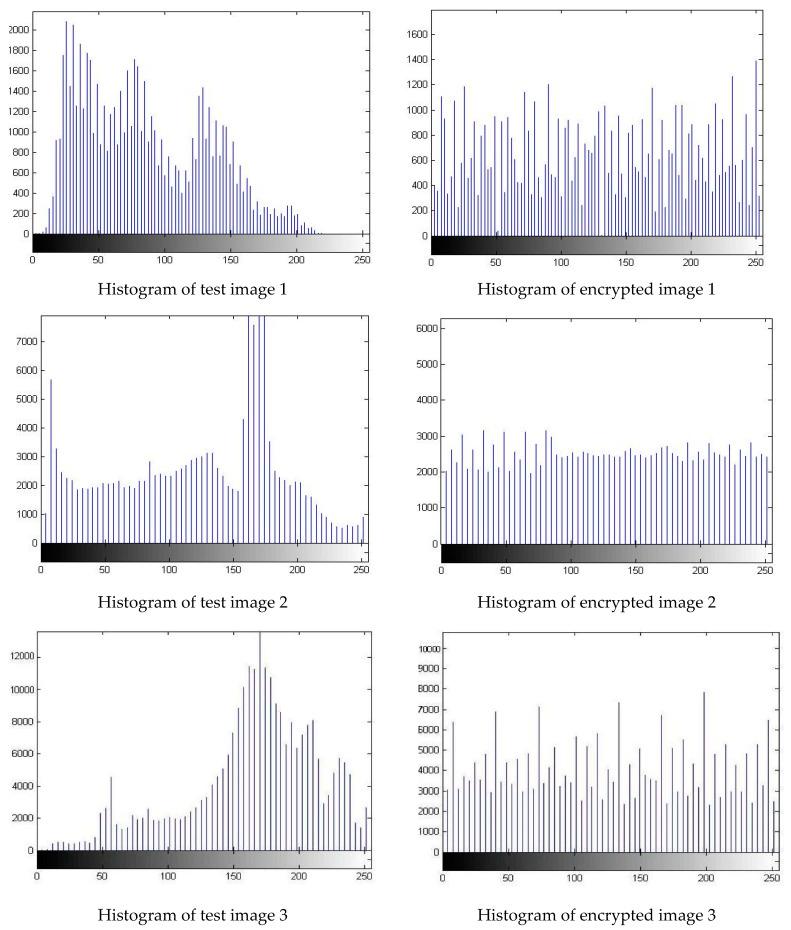

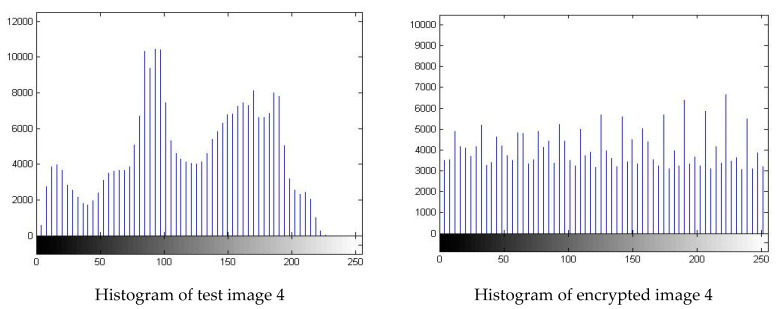
Statistical analysis of the proposed algorithm using histograms of four images. The histograms of the test images are shown in the first column and that of the encrypted images are shown in the second column. It can be seen that the histograms of the encrypted images approach uniform histograms.

The statistical analysis of the proposed approach using a histogram is presented in Figure 8. The histograms of four test images and their encrypted versions are illustrated in Figure 8. From this Figure 8, it is evident that the histograms of the encoded image are quite different from that of the test images. The histograms of the encrypted images resemble the uniform distribution. The shape of the histogram of the encrypted image is nearly constant and notably different from that of the test image. This shows that the algorithm is capable of mitigating statistical attacks.

#### 3.1.2. Correlation Analysis

Correlation is a statistic that measures the degree to which two entities correlate to each other. It is widely used in image processing research for investigating the similarity between two images. The value of correlation ranges between −1 and 1. A higher value of correlation means more similarity between the two images. It is used as a metric to measure the robustness of the approach toward statistical attacks. An ideal encryption algorithm produces encrypted images such that the correlation between the test image and the encrypted image is equal to zero, expressing that the test image and the encrypted image are almost independent. Similarly, the algorithm reproduces the decrypted images such that the correlation between the test image and the reconstructed image is equal to one, which means a perfect reconstruction.

The correlation analysis explores the relationships between nearby pixels in the test image and the encrypted image. A higher correlation between the adjacent pixels indicates a higher sensitivity to statistical attack. Good encryption algorithms tend to reduce the coefficient value. In this paper, the following equations are used to calculate the correlation coefficients $r_{x,y}$ of each pair:

(3) $$r_{x,y}=\frac{E(\left(x-E\left(x\right)\right)\left(y-E\left(y\right)\right)}{\surd D\left(x\right)D\left(y\right)}$$

(4) $$E\left(x\right)=\frac{1}{N}\sum_{i=1}^{N}x_{i}$$

(5) $$D\left(x\right)=\frac{1}{N}\overset{N}{\underset{i=1}{\sum }}{\left(x_{i}-E\left(x\right)\right)}^{2}$$

Here, the expectations and variance of the variable *x* are denoted as *E*(*x*) and *D*(*x*), respectively.

**Figure 9:**
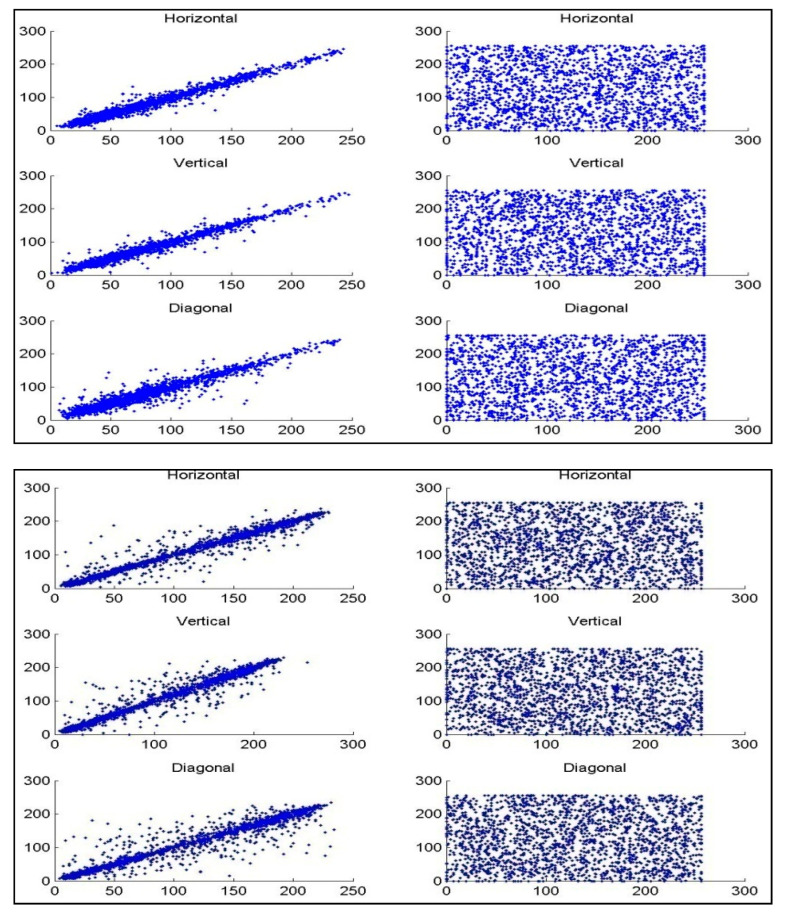
Statistical analysis of the proposed algorithm using correlation coefficients. The correlation between the test image and the decrypted image is shown in the first column and that between the test and encrypted images is shown in the second column. The test image and encrypted image are highly uncorrelated, whereas the test and decrypted image are highly correlated.

**Figure 10:**
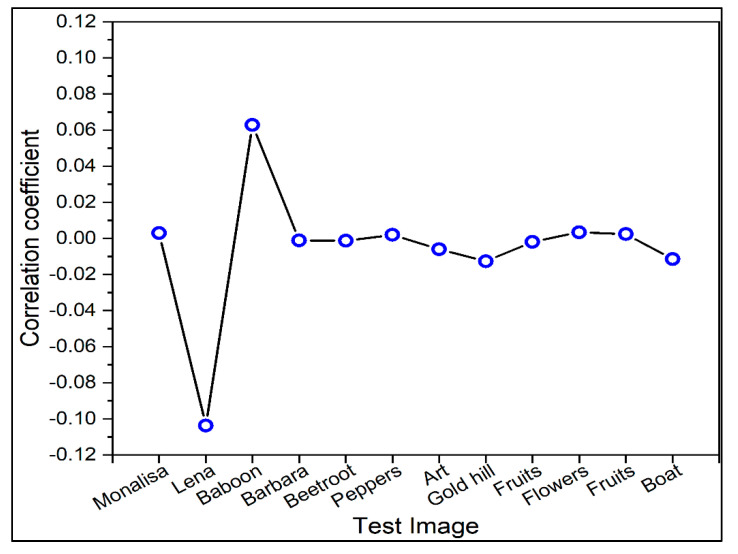
Correlation coefficients analysis for different test images.

To carry out the correlation analysis, 3000 pairs of adjacent pixels from the first image and the encoded image are randomly selected in the level, vertical, and corner-to-corner headings. Figure 9 illustrates the correlation coefficients between the test image and the encrypted image for the ‘Lena’ test image. The correlations between two nearby pixels are shown in three different ways, namely horizontal, vertical, and diagonal. The correlation between the adjacent pixels in the test image is moderately high, which could result in some sequence seepage, while it is extremely low in the encrypted image. The analysis of the correlation coefficients of the 12 test images is presented in Figure 10. It can be seen that the correlation coefficients of 10 test images lie between −0.02 and 0.02. The correlation coefficient for the ‘Lena’ image and ‘Baboon’ images are approximately −0.1 and 0.06, respectively. The correlation coefficients of all 12 images are lower than 0.10. Since the correlation between the test image and encrypted images has been successfully eliminated, it can be claimed that the proposed approach is robust against statistical attacks.

**Figure 11:**
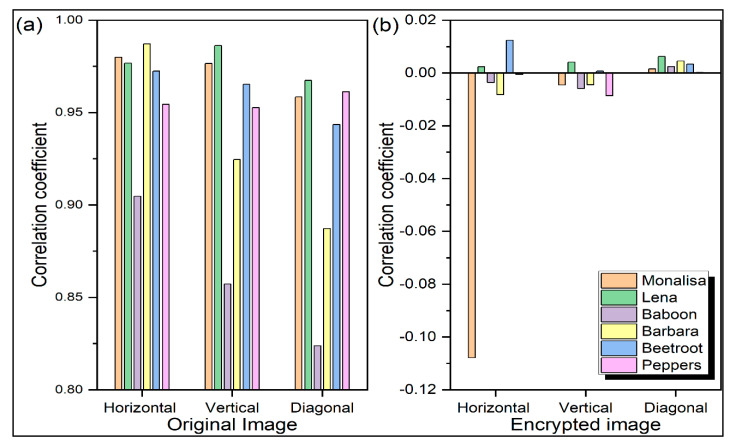
Correlations of two adjacent pixels in input and output images in horizontal, vertical, and diagonal directions. (**a**) Original image; (**b**) Encrypted image.

The correlation of two adjacent pixels for six images, namely ‘Monalisa’, ‘Lena’, ‘Baboon’, ‘Barbara’, ‘Beetroot’, and ‘Peppers’ is shown in Figure 11. Here, three directions, namely, horizontal, vertical, and diagonal are considered for input and output images. From Figure 11, it is clear that the correlation of two adjacent pixels in all test images is nearly one, whereas that of encrypted images is near zero. The results indicate that the correlation between adjacent pixels in the images has also been successfully eliminated. These results support the robustness of the algorithm against statistical attacks.

### 3.2. Differential Analysis

One of the characteristics of a good encryption algorithm is that any tampering with the test image must result in a substantial change in the encrypted image. In other words, even a one-bit change in the test image should generate a different encrypted image. Attackers attempt to derive the encryption key by finding a relationship between the test image and the encrypted image. For this, they analyse how modifications in a test image affect the resultant difference in the encrypted image. Differential attack analysis is used to assess the variations in the encrypted image after making a minute change in a pixel of the test image. The suggested approach is used to encrypt the test image before and after the slight modification to the test image that is created as part of the discrepancy harass.

**Figure 12:**
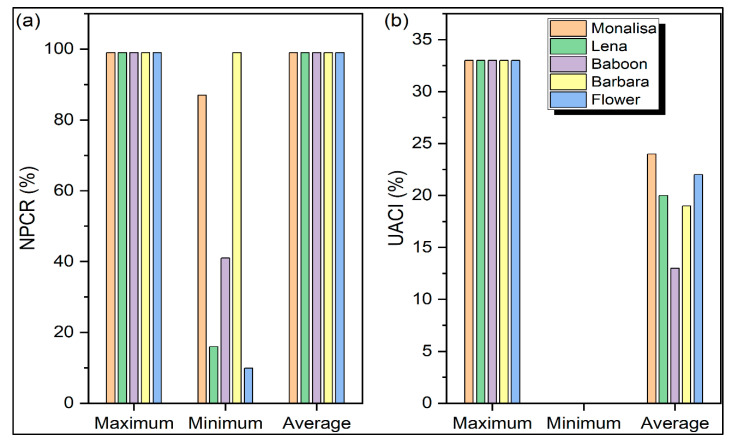
Analysis of security of several encryption techniques using (**a**) number of pixels changing per second (*NPCR*) and (**b**) unified average changing intensity (*UACI*).

Every possible link between the test image and the encrypted image has been found between these two encrypted images. Researchers often employ two criteria to evaluate the differential attack resistance of any encryption algorithm: the Unified Average Changing Intensity (*UACI*) and the Number of Pixels Change Rate (*NPCR*). The *UACI* represents the average intensity of the difference between pixels at the same positions in test images, before and after the differential attack. The *NPCR* means the percentage of different pixels at the same position between two corresponding encrypted images which are obtained by two images with a one-bit difference. The *NPCR* and *UACI* are shown as Equations (6) and (7),

(6) $$NPCR=\frac{\sum_{i=1}^{M}\sum_{j=1}^{N}D\left(i,j\right)}{MxN}\times 100\%$$

(7) $$UACI=\frac{\sum_{i=1}^{M}\sum_{j=1}^{N}\left|c_{1\;}\left(i,j\right)-c_{2}\left(i,j\right)\right|}{255\times M\times N}\times 100\%$$

(8) $$\mathrm{Subject}\;\mathrm{to},\;D\left(i,j\right)=\left\{\begin{array}{c} 0\;\;\;\;ifc_{1}\left(i,j\right)=c_{2}\left(i,j\right)\\ 1\;\;ifc_{1}\left(i,j\right)\neq c_{2}\left(i,j\right) \end{array}\right.$$

where *M* and *N* stand for the image’s height and breadth, respectively. Two encrypted photos, *C*_1_ and *C*_2_, have a pixel difference. The minimum, maximum, and average *NPCR* and *UACI* values for five test images, namely, ‘Monalisa’, ‘Barbara’, ‘Lena’, ‘Baboon’, and ‘Flower’, are displayed in Figure 12. The maximum and average values of the *NPCR* for all images are 100. Similarly, the maximum values of the *UACI* for all images are nearly 35, whereas the minimum value of *UACI* for all images is 0. The minimum *NPCR* values of all the test images vary between 15 and 100. The average *UACI* values of all the images approaches 20. This analysis proves the efficacy of the proposed approach against differential attacks.

### 3.3. Encryption Time Analysis

**Figure 13:**
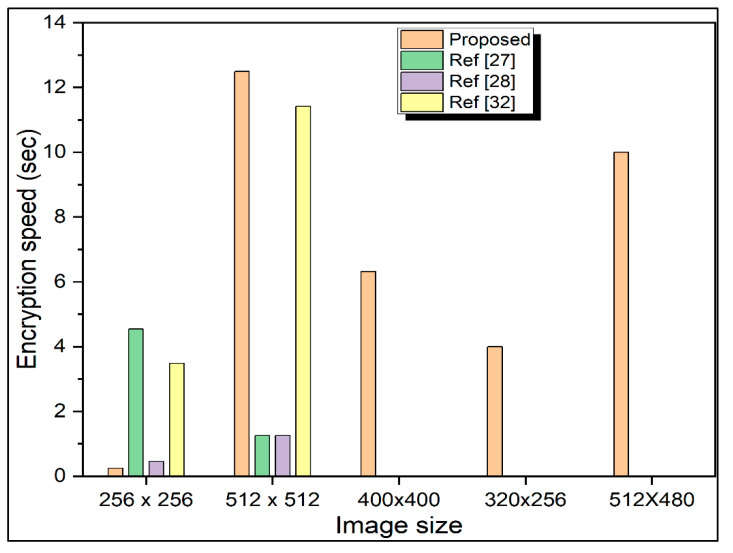
Encryption time analysis and comparison with other methods for images of different sizes.

When the security level may match the criteria, running encryption time is an important characteristic parameter for encryption algorithms. Crossover and mutation processes are the two main components of genetic-based picture encryption schemes. The suggested approach uses a hybrid mutation mechanism that involves block swapping. Figure 13 illustrates the encryption time analysis of the proposed approach for test images of different sizes. It can be seen from Figure 13 that computational complexity increases with the size of the image. The time complexity of the approach is moderate in most cases but sufficient for real-time communication in the case of small-size images. Figure 13 also compares the performance of the proposed approach in terms of encryption time analysis with that of similar methods proposed in [19,46,47]. It can be noticed that the current approach outperforms all the methods in terms of computational complexity for test images of the size 512 × 512 × 3.

### 3.4. Information Entropy

Information entropy is a measure of randomness in signals. The value of information entropy ranges from 0 to 1. If the information is more regular, the information entropy will be smaller. If the information entropy is 1, it means the signal has a high degree of randomness. The confidentiality of test images and encrypted images can be described by the value of information entropy. The greater the information entropy, the better the confidentiality. The information entropy able to be designed by,

(9) $$H(m)=\sum_{i=0}^{2^{n}-1}x\left(m_{i}\right)log\frac{1}{x\left(m_{i}\right)}$$

where H(*m*) represents the information entropy of a sequence source *m* and *x*(*mi*) denotes the probability of symbol *m_i_*. The entropy should be eight times higher for a chaotic image with 256 shades of grey.

**Figure 14:**
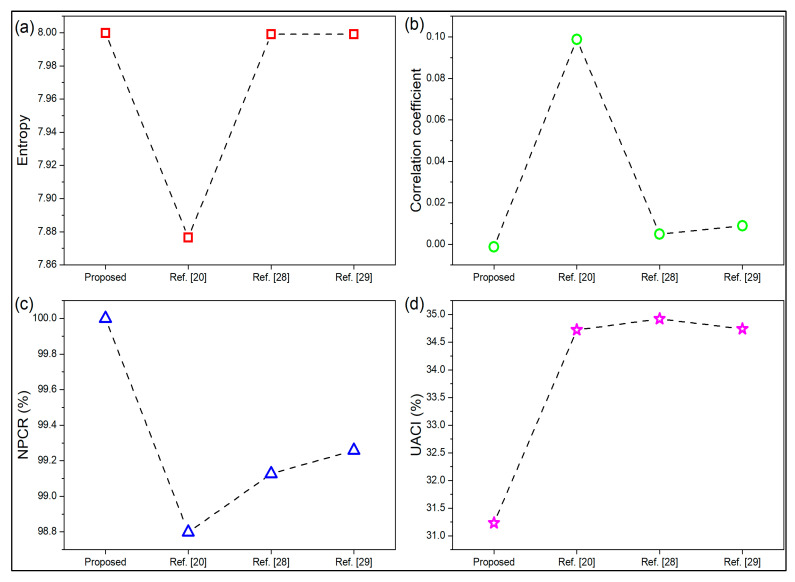
Performance comparisons of the proposed scheme with three other methods proposed in the literature for images of the size 512 × 512. (**a**) Entropy; (**b**) Correlation coefficient; (**c**) *NPCR*%; (**d**) *UACI*%.

The overall performance comparison of the proposed scheme with that of the other three methods reported in [16,19,20] for images of the size 512 × 512 is presented in Figure 14. The comparison is illustrated in terms of entropy, correlation coefficient, *NPCR*, and *UACI*. It reveals that the proposed approach performs at par with the existing methods with a speed advantage for an image of a larger size.

**Figure 15:**
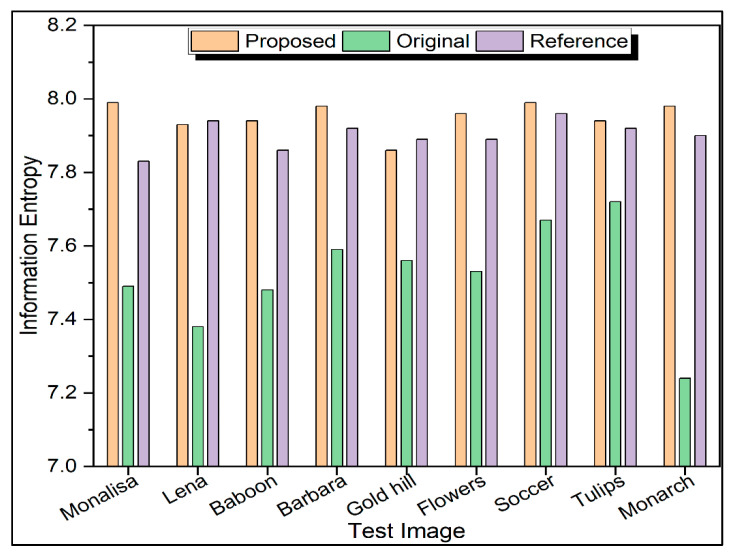
Information entropy of input and encrypted image.

The information entropy of nine test images and corresponding encrypted images is displayed in Figure 15. The information entropies produced by the current method are almost identical to those reported in previous works in the literature [19,20] and closer to the theoretical value of 8 for the majority of the test images.

### 3.5. Robustness Analysis

#### 3.5.1. Chosen Plain Image Attack Analysis

**Figure 16:**
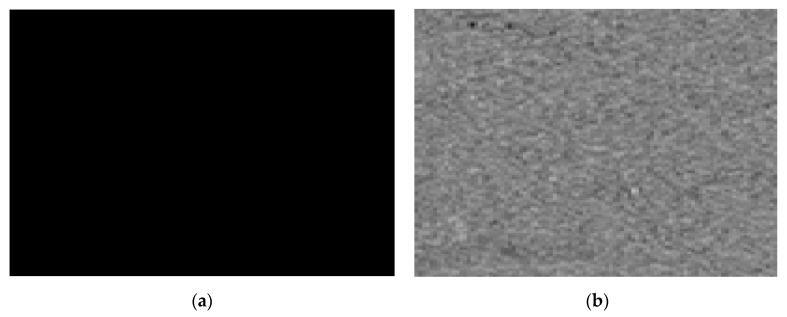

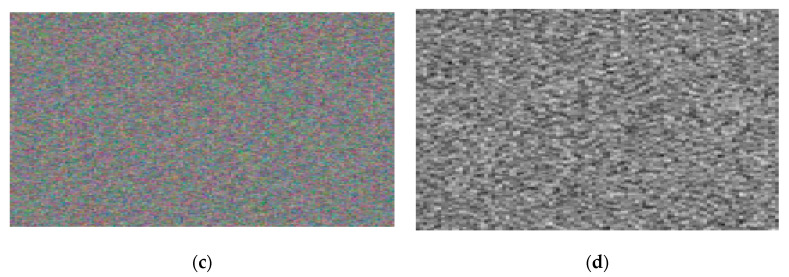
The results of the chosen plain image attack. (**a**) Black image; (**b**) Encrypted black image; (**c**) Encrypted original image; (**d**) Decryption of encrypted black image with possible key.

Attackers typically choose plain images, such as black images, for chosen plain image attacks. It removes the typical image attributes from the method and the encryption key because its pixel value is zero. In Figure 16, the encrypted image displays the outcomes of our chosen plain image attack using the black image. The cryptanalyst takes this data as a potential key and makes an effort to decrypt any other passwords that might have been encrypted using the key. The results indicate that no meaningful information can be gained in Figure 16 after using the potential information to decrypt the original image. As a result, the chosen attack will not succeed against our proposed methodology.

#### 3.5.2. Occlusion Attack Analysis

**Figure 17:**
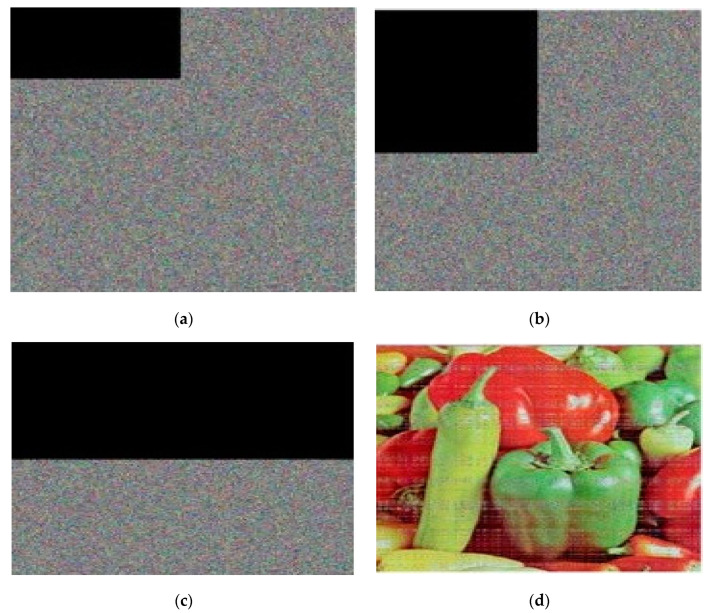

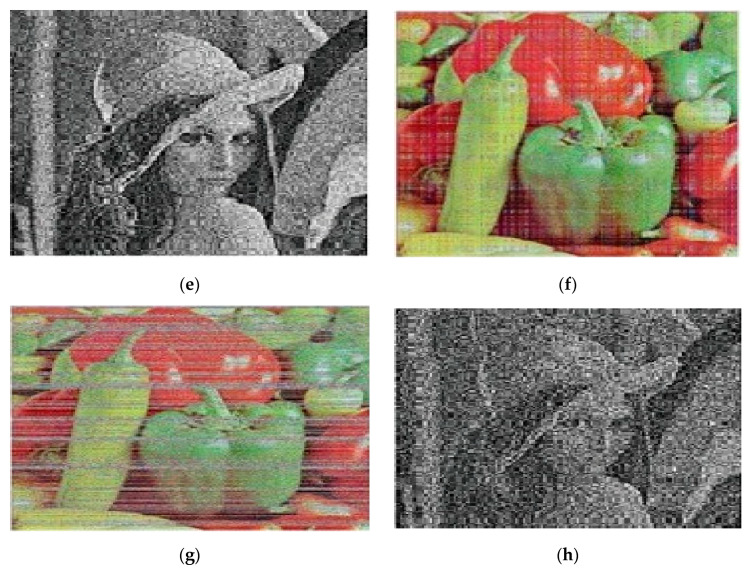
The results of occlusion attack. (**a**) Cipher image with 12.5% occlusion; (**b**) Cipher image with 25% occlusion; (**c**) Cipher image with 50% occlusion; (**d**) Decrypted image with 12.5% occlusion; (**e**) Decrypted image with 25% occlusion; (**f**) Decrypted image with 25% occlusion; (**g**) Decrypted image with 50% occlusion; (**h**) Decrypted image with 50% occlusion.

In an occlusion attack, we select 12.5%, 25%, and 50% of the occlusion in an encrypted image. Figure 17 displays the attack’s results. The findings demonstrate that the suggested cryptographic technique may successfully resist occlusion attacks.

#### 3.5.3. Noise Attack Analysis

**Figure 18:**
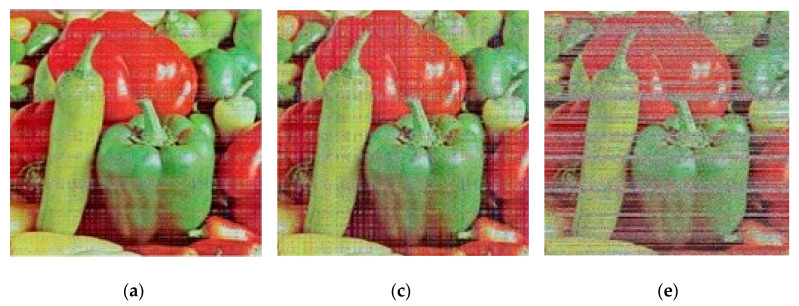

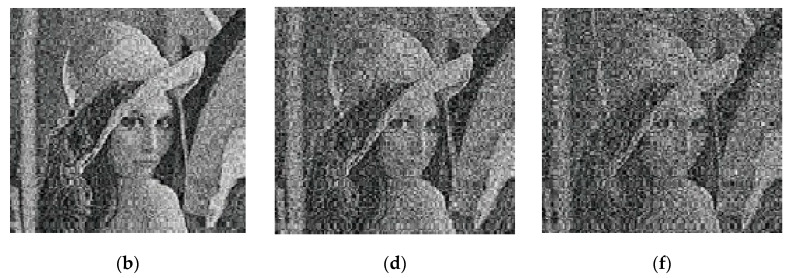
The results of noise attack. (**a**) Noise with 10 intensity; (**b**) Noise with 10 intensity; (**c**) Noise with 15 intensity; (**d**) Noise with 15 intensity; (**e**) Noise with 20 intensity; (**f**) Noise with 20 intensity.

The proposed algorithm’s antinoise performance was tested by adding varying intensities of Gaussian noise to the encrypted image. They were then decrypted, and the intensities were 10, 15, and 20, respectively. The results are displayed in Figure 18. As can be seen, once the noise image has been encrypted, the original image may essentially be reconstructed. As a result, the proposed methodology has some noise attack resistance.

### 3.6. Comparision with Previous Studies

The suggested cryptosystem is contrasted with the existing image encryption techniques based on specific execution pointers, as referred to in [7,16,19,20,47,48]. Despite not being the largest, this plan’s room for crucial space examination is sufficiently large to fend off a thorough attack. The current cryptosystem’s correlation coefficients are closer to 0 than the encryption methods [7,16,19,20,47,48], which reveals that the cryptosystem is more resistant to factual attacks. In contrast to those in several sources in the literature [7,16,19,20,47,48], the data entropy in this article is larger. The proposed cryptosystem’s *NPCR* and *UACI* estimates are very close to the ideal values, making it an image cryptosystem that can withstand known-plaintext and selected plaintext attacks.

Table 1 compares the current framework to the one that is currently in place. As a result, the suggested image cryptosystem’s viability and feasibility are supported.

## 4. Conclusions and Discussion

This research proposed a genetic operator-based image encryption technique. At the start of the current procedure, the test image is divided and blended. Then, a bitplane slicing technique is used to divide the scrambled image into eight bitplanes. The blocks of each bitplane are randomly exchanged before crossover and mutation procedures. Block switching, crossover mutation, and hybridisation are employed to boost the system security. Multiple bitplane decompositions can operate simultaneously according to the provided approach. Numerous tests are run to verify the system’s security and operation. It is possible to adapt the given algorithm to include the concepts of video encryption by using confusion and diffusion techniques. The chaotic character of the higher dimensional chaotic maps remains to be theoretically analysed. The development of a general selective encryption technique for both still images and moving pictures that satisfy the aforementioned desirable objectives is a difficult task. Ideally, our findings for image protection can be applied to more computationally intensive video protection. It is possible to look at rich hardware implementation platforms.

## Figures and Tables

**Table 1:** Statistical comparison with existing works.

Algorithm	Information Entropy	Correlation Coefficient	PSNR	*NPCR* (%)	*UACI* (%)	Robustness Analysis	Speed
Proposed	7.9999	−0.001272	49.23	99.9992	31.23	Yes	0.2456
Ref. [7]	7.9998	0.006532	48.16	99.9914	33.54	No	0.4869
Ref. [16]	7.8765	0.098747	44.52	98.7982	34.72	No	0.7865
Ref. [19]	7.9992	0.004875	44.49	99.1265	34.92	No	0.7025
Ref. [20]	7.9991	0.008956	48.10	99.2584	34.74	No	0.5812
Ref. [47]	7.9995	0.000125	47.74	99.2496	34.86	No	0.5716
Ref. [48]	7.8992	0.000862	45.23	98.6514	34.86	No	0.7528
Ref. [47]	7.9987	0.004532	48.23	99.5424	35.21	No	0.6423
Ref. [48]	7.9954	0.002354	49.16	99.4625	34.78	No	0.6845

## Data Availability

The data presented in this study are available through email upon request to the corresponding author.
